# Vertical Movement Patterns and Ontogenetic Niche Expansion in the Tiger Shark, *Galeocerdo cuvier*

**DOI:** 10.1371/journal.pone.0116720

**Published:** 2015-01-28

**Authors:** André S. Afonso, Fábio H. V. Hazin

**Affiliations:** 1 Departamento de Pesca e Aquicultura, Universidade Federal Rural de Pernambuco, Recife, Pernambuco, Brazil; 2 Faculdade de Ciências e Tecnologia, Universidade do Algarve, Faro, Portugal; Aristotle University of Thessaloniki, GREECE

## Abstract

Sharks are top predators in many marine ecosystems and can impact community dynamics, yet many shark populations are undergoing severe declines primarily due to overfishing. Obtaining species-specific knowledge on shark spatial ecology is important to implement adequate management strategies for the effective conservation of these *taxa*. This is particularly relevant concerning highly-mobile species that use wide home ranges comprising coastal and oceanic habitats, such as tiger sharks, *Galeocerdo cuvier*. We deployed satellite tags in 20 juvenile tiger sharks off northeastern Brazil to assess the effect of intrinsic and extrinsic factors on depth and temperature usage. Sharks were tracked for a total of 1184 d and used waters up to 1112 m in depth. The minimum temperature recorded equaled 4°C. All sharks had a clear preference for surface (< 5 m) waters but variability in depth usage was observed as some sharks used mostly shallow (< 60 m) waters whereas others made frequent incursions into greater depths. A diel behavioral shift was detected, with sharks spending considerably more time in surface (< 10 m) waters during the night. Moreover, a clear ontogenetic expansion in the vertical range of tiger shark habitat was observed, with generalized linear models estimating a ~4-fold increase in maximum diving depth from 150- to 300-cm size-classes. The time spent in the upper 5 m of the water column did not vary ontogenetically but shark size was the most important factor explaining the utilization of deeper water layers. Young-of-the-year tiger sharks seem to associate with shallow, neritic habitats but they progressively move into deeper oceanic habitats as they grow larger. Such an early plasticity in habitat use could endow tiger sharks with access to previously unavailable prey, thus contributing to a wider ecological niche.

## Introduction

Sharks are generally acknowledged as important elements in many marine ecosystems because they usually occupy high levels in trophic webs [1], thus they may regulate the abundance and behavior of lower *taxa* through predator-prey interactions [2–4]. Also, they have proven to be susceptible to overexploitation [5, 6] and habitat degradation [7, 8] due to typical K-selected life-history strategies such as slow growth, late maturity and low fecundity [9, 10]. Nevertheless, many shark populations experience severe fishing pressure worldwide [11, 12] and historical abundance declines have been reported for some exploited species [13–15]. The depletion in shark abundance may lead to serious environmental damage through mesopredator releases and trophic cascades [4, 16–19], therefore the conservation of their populations is warranted.

Understanding the spatiotemporal distribution of shark populations within their range is important for the optimization of fisheries management strategies and conservation measures. Surveying the relative abundance of species with adequate fishing gear may provide a proxy of their distribution, however in most circumstances such an approach could be insufficient due to intrinsic sampling bias from typically uncontrolled factors, particularly if fisheries-independent data are not available [20–22]. Such tasks are further complicated when addressing species with large home ranges in the oceanic realm. For these sharks, measuring individual movements and extrapolating trends at population-level could be an important complementary tool. The spatial distribution of sharks is influenced by species-specific activity and behavior patterns associated with motivational and energetic requirements, which ultimately determine essential traits such as foraging strategies and encounter rates with prey, the location of mates and timing of courtship, and habitat preferences [23]. Spatial ecology is thus a key-component for the effective management and conservation of sharks because individual movements will regulate the dynamics in the population distribution.

The spatiotemporal dynamics of shark distribution and behavior are complex, involving distinct processes acting at different scales such as daily and seasonal migrations, regional variability in habitat preferences, and segregation by age or gender [24]. Increasing our understanding of the patterns of habitat use, movement, and behavior should contribute to defining spatiotemporally-integrated management measures, which could promote the sustainability of fisheries and the conservation of species more efficiently [25]. On that account, assessing the vertical component (i.e., depth) of shark movements is particularly useful for deriving depth and thermal niches and assessing behavioral shifts across some environmental scale. Pop-up satellite archival tags (PSAT) have proved to be effective in tracking vertical movements [26–28] and several studies have deployed these tags on sharks [29]. Also, vertical movement analysis could be relevant for understanding the processes that regulate habitat shifting in species with both coastal and oceanic distributions, since the continental platform imposes bathymetric constraints to shark movements that could be differentiated from movements performed in deep waters from the oceanic realm. The tiger shark, *Galeocerdo cuvier*, is one of such species, occurring in the neritic as much as in the oceanic provinces from tropical and warm-temperate latitudes worldwide [30].

Tiger sharks are among the most abundant shark species off Recife, northeastern Brazil [31] and have large home ranges as they move through considerable distances in nearshore and oceanic waters [32, 33], inclusively across oceanic basins [34, 35]. Although tiger sharks do not use specific nursery areas as many other carcharhinids do [36], there is evidence from the North Atlantic Ocean that they could use discrete locations in the continental shelf as primary pupping areas, where parturition occurs and from where neonates disperse [37]. Consequently, juvenile tiger sharks would first occupy neritic habitats before moving to oceanic areas. This seems to be confirmed by the fact that individuals measuring < 200 cm TL comprise the bulk of the tiger shark catch in nearshore waters off Recife [31]. As tiger sharks grow larger they undergo ontogenetic dietary shifts which result in a higher diversification of prey [38, 39]. Juvenile dispersion into oceanic waters could further contribute to a more generalist feeding behavior as it would allow access to different prey items that are unavailable in coastal areas. On the other hand, cyclical patterns derived from diel and seasonal rhythmicity may also have an effect on tiger shark habitat use and behavior [40–42]. The effective conservation of tiger shark populations could depend on all these processes being adequately incorporated into fisheries management frameworks because features such as the depth of the fishing gear and the spatiotemporal distribution of fishing effort expectedly have a great influence on the catch rate of sharks.

In this context, this study aims at assessing vertical movement patterns and thermal preferences of juvenile tiger sharks in the southwestern equatorial Atlantic Ocean, and testing the effect of some intrinsic (i.e. shark size and sex) and extrinsic (i.e. diel and lunar cycles) factors on the spatial ecology of this species.

## Material and Methods

### Ethics statement

This study has been conducted in accordance with Brazilian regulations for wildlife research and it was approved by the Instituto Chico Mendes de Conservação da Biodiversidade of the Brazilian Ministry of the Environment (permit no. 15083-8). Tiger shark capture, handling and tagging was approved and carried out in full compliance with the recommendations of the Regiment of the Commission of Ethics on the Usage of Animals from the Universidade Federal Rural de Pernambuco (license no. 041/2009; protocol no. 23082.009679/2009 D18). No endangered or protected species were involved in this study.

### Shark capture and tagging

Tiger sharks were caught with bottom longlines and drumlines deployed off Recife (8°10′S, 34°53′W), northeastern Brazil, between June 2008 and September 2012. Longlines were equipped with 17/0 circle hooks and were allowed to fish overnight, with typical soak time equaling 14–15 hours [31]. All sharks were brought onboard, carefully accommodated in a tank filled with running seawater, eye-covered with a soaked dark tissue and, if the shark had been caught in nearshore waters, transported seaward to deeper isobaths. For translocated sharks, the time spent aboard ranged between 30 and 90 minutes. The need to translocate sharks from shallow to deeper waters derived from the shark attack mitigation strategy of the Shark Monitoring Program of Recife [31], which aims at removing potentially aggressive sharks from hazardous areas following a sudden increase in the shark attack rate on humans in this region [43]. All sharks were sexed, measured for stretched total length (TL) to the nearest centimeter, and tagged with a pop-up satellite archival tag (PSAT). PSAT-tag models were either mk10-PAT or miniPAT (Wildlife computers, WA). These tags archive depth and temperature readings during deployment and self-release from the shark after a user-programmable amount of time in order to stream summaries of the archived data to the ARGOS satellites. PSAT tags were rigged with a 2.0-mm polyamide monofilament coated with high-resistance spectra braid and dark heat-shrinking tubing. Tags were fitted to the sharks by passing the coated monofilament through a ~3 mm puncture at the proximal middle portion of the first dorsal fin and crimping it tight with a stainless steel sleeve so that the tag would be towed just behind the first dorsal fin. This attachment method was feasible regardless of oceanographic conditions and allowed to keep invasive procedures to a minimum.

### PSAT tag programming

PSAT tags were programmed to record water depth (±0.5 m) and temperature (±0.05°C) every second for a period between 30 and 180 days and to summarize the data into temporal bins ranging from 2 to 24 hours for satellite data streaming (Table 1). For mk10 tags, depth and temperature data were binned into 14 user-customized strata and the time spent at each stratum was recorded. Disregarding minor variations in the first deployments, depth strata were arranged in classes < 1, 1−5, 5−10, 10–20, 20–40, 40–60, 60–80, 80–100, 100–125, 125–150, 150–200, 200–250, 250–300, and > 300 m, while temperature strata were arranged in classes < 12, 12–14, 14–16, 16–18, 18–20, 20–22, 22–24, 24–25, 25–26, 26–27, 27–28, 28–29, 29–30, and > 30°C. MiniPAT tags allowed time-at-depth (TAD) and time-at-temperature (TAT) data to be arranged in 12 strata only, but strata limits were set to match those from mk10 tags to allow comparisons involving different tag types. Furthermore, heterogeneous stratum sizes required TAD data to be standardized by depth-unit (i.e., translated into time-per-unit-of-depth, TPUD) for inspecting tiger shark preferences for a specific depth. While this approach assumes that sharks use all available depths within a depth stratum equally, which may not always be true, failing to standardize TAD data before interpreting tiger shark environmental preferences could be misleading because wider strata will have an increased importance even if sharks hypothetically make random use of the whole water column.

**Table 1:** Summary of tag deployments on 20 tiger sharks off northeastern Brazil.

**Shark**	**Sex**	**TL**	**DepDate**	**TDate**	**PopLoc**		**D (d)**	**P (d)**	**R (h)**
					Lat	Lon			
T1	M	131	28 Jun 2008	28 Jul 2008	−6.33	−34.80	30	30	24
T2	M	193	24 Jul 2009	31 Jul 2009	−6.62	−34.80	7	74	3
T3	F	128	1 Jun 2010	13 Aug 2010	−7.98	−34.67	74	74	3
T4	F	154	1 Aug 2010	11 Sep 2010	**	**	42	50	2
T5	M	150	7 Aug 2010	23 Out 2010	−7.08	−34.85	78	99	3*
T6	F	190	21 Dec 2010	16 Jan 2011	−4.77	−35.90	29	48	3
T7	F	295	6 Jan 2011	12 Apr 2011	−3.65	−37.2	98	96	4
T8	M	190	8 Feb 2011	3 Mar 2011	−3.33	−37.46	27	49	3
T9	F	120	5 Mar 2011	14 May 2011	−4.63	−35.20	70	70	6
T10	F	270	22 Mar 2011	1 Sep 2011	−2.69	−42.06	159	120	12
T11	M	125	11 Jul 2011	25 Aug 2011	−7.02	−34.84	47	100	3*
T12	M	170	11 Jul 2011	9 Sep 2011	−12.57	−37.91	61	60	4
T13	F	134	25 Jul 2011	15 Sep 2011	−4.70	−35.75	53	62	12
T14	M	156	14 Aug 2011	13 Oct 2011	−6.95	−34.81	59	60	4
T15	F	172	23 Aug 2011	2 Sep 2011	−5.18	−35.40	14	60	4
T16	F	253	5 Sep 2011	18 Oct 2011	−11.80	−33.79	42	180	12
T17	F	300	5 May 2012	10 Jun 2012	**	**	36	60	4
T18	F	180	24 Aug 2012	25 Sep 2012	−17.25	−37.10	33	60	4
T19	F	156	9 Sep 2012	10 Jan 2013	−18.50	−36.55	123	120	6
T20	M	170	17 Sep 2012	27 Dec 2012	−3.87	−38.46	102	120	3*

Included is the shark number and sex, total length in centimeters (TL), deployment date (DepDate), date of first transmission (TDate), pop-up location (PopLoc), track duration in days (D), programmed track duration in days (P), and temporal resolution of summarized data in hours (R). Sharks were tagged off Recife (about 8.2°S and 34.8°W).

*recovered tags

**no geolocation data available

M:male, F:female

### Statistical analyses

Prior to analyses, the first week of data after tagging was discarded to minimize potential bias due to unnatural post-release behavior [44]. Also, high-resolution archival data from recovered PSAT tags were pooled in 3-hour bins to match the temporal resolution of ARGOS-relayed datasets of other tags. The vertical distribution of tiger sharks across the study period and across diel and lunar cycles was plotted, together with depth-temperature profiles of the water column. To conduct a first inspection for a possible influence of sex and size on tiger shark vertical distribution, male and female sharks were classified as small (< 150 cm TL), medium (≥ 150 and < 250 cm TL), and large (≥ 250 cm TL) specimens for analytical convenience and Kruskal-Wallis rank sum tests were performed using maximum diving depth (MDD) and minimum diving temperature (MDT) as the response variables. Comparisons between sexes were conducted using four medium-sized sharks with similar lengths to prevent incorporating variability resulting from shark length into the model.

The effect of a set of biological and environmental factors on the vertical distribution of tiger sharks was further investigated with generalized linear models (GLM). Explanatory variables were TL (continuous), sex (2-level factor), diel cycle (2-level factor) and lunar cycle (4-level factor), whereas response variables were MDD, MDT and time at surface (TAS), i.e. the proportion of time spent at a surface water-layer of a given width. TAS modeling involved 7 distinct approaches across an increasing depth range, with surface being interpreted as the topmost layer of the water column between the sea surface and the 5-, 10-, 20-, 40-, 60-, 100- and 150-m isobaths. Prior to these analyses, the dataset of each shark was aggregated into 12-h periods so that each tracking day had two samples matching the diel cycle, i.e 18:00h-06:00h and 06:00h-18:00h. Such conservative time-window was selected in order to overcome any possible autocorrelation in depth and temperature data. Since tiger sharks frequently swim in an yo-yo diving pattern with relatively short periods [44, 45] and have high affinity for surface waters [35, 46], they repeatedly transverse a wide portion of the water column in relatively little time. Therefore, the response variables herein considered are expectedly independent using a 12-h denominator. Eligible tracks comprised tags with data summarization periods of 12 h or less. Because tag deployments provided different amounts of data and sharks with more data could bias the analyses, the average number of samples per shark was calculated and data of sharks that had more samples than the average were sub-sampled randomly so that the number of samples would match the average number of samples per shark.

The most adequate error distribution and canonical link function were assessed separately for MDD, MDT and TAS models. The Gaussian and Gamma error distributions with identity, logarithmic, square root and inverse link functions were compared for MDD and MDT models, whereas the Binomial and Quasi-binomial error distributions with logit, cauchit, probit and cloglog link functions were compared for TAS models. Model diagnosis for each combination of error distribution and canonical link function were performed and the combinations that showed best residual performance were selected. For TAS models, the error distribution and link function which generally performed best were used in the 7 different approaches for coherence sake. Model-building followed a forward stepwise approach and the Akaike information criterion (AIC) was used to select the best model. The statistical significance of regression coefficients was assessed with Wald tests and the inclusion of selected variables in the model depended on their significance. At each step, the significance of the amount of variability explained by the inclusion of additional variables into the model was tested with likelihood ratio tests and the new model was discarded if not significant (α = 0.05). Residual diagnostic plots were assessed for the final models to ensure they met their assumptions. All statistical analyses were performed in *R* version 2.14.0 (*R* Development Core Team 2011).

## Results

A total of 20 tiger sharks measuring (mean ± standard deviation) 181.8 (± 55.3) cm TL (range = 120–300) were tagged and released off Recife (Table 1). The sex ratio was female-biased and equaled 1.5:1. Despite that translocated sharks endured prolonged restraining on the deck of the vessel, resulting in a few of those sharks being assisted by scientific personnel upon release due to unresponsive behavior, no episodes of post-release mortality have been detected [44]. Nevertheless, premature releases amounted to 60% of deployed tags (Table 1), one of which, i.e. shark T13, was ascribed to an episode of natural mortality [44]. Altogether, tiger sharks were tracked for a total of 1184 d, ranging from 4 to 159 d and averaging 59.2 (± 38.1) d per shark. This corresponded to 74% of the overall programmed study span. Satellite data streaming yielded 11347 successfully decoded messages, averaging 597 (± 658) messages per tag. Two prematurely-released tags, i.e. T4 and T17, failed to report any useful data. Also, three tags were physically recovered either by fishermen or after washing ashore, thus providing the complete archived dataset. Most tags popped-off to the north of Recife in both coastal and oceanic waters but a few tags popped-off to the south, usually in oceanic waters [44].

### Depth and temperature distribution

**Figure 1:**
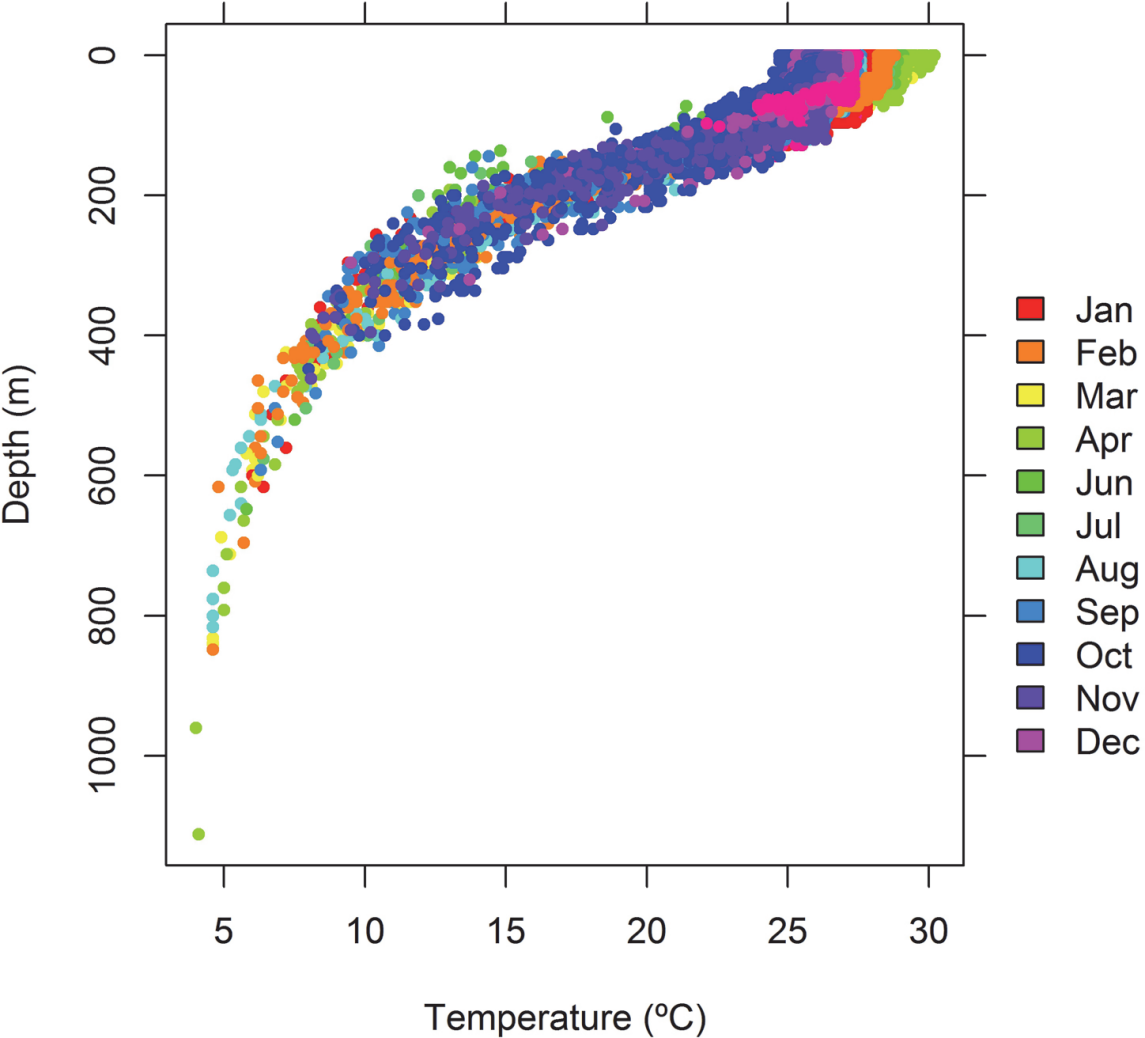
Vertical profile of the seawater temperature. Depth-and-temperature data sampled by tiger sharks off northeastern Brazil. Colors represent months of the year to depict temperature seasonality in the mixed surface layer, which extends to about the 60-m isobath.

Tiger sharks in this study reached an average maximum depth of 449 (± 257) m, with the deepest record equaling 1112 m (Table 2). As a result, they experienced a wide thermal gradient, with minimum temperatures ranging from 4.0 to 25.0°C and averaging 9.7°C (± 4.8°C), maximum temperatures ranging from 26.6 to 31.2°C and averaging 28.1°C (± 1.3°C), and temperature amplitudes averaging 18.3°C (± 5.3°C) and being as wide as 27.2°C (Table 2). The vertical distribution of temperature data pooled for all sharks revealed a mixed surface layer (MSL) extending to about 60 m in depth and a broad thermocline occupying further depths until the 500–600 m isobaths, across which temperature dropped nearly 20°C (Fig. 1). Temperature in the MSL was apparently seasonal, being highest in March-April and lowest around October.

**Table 2:** Summary of depths and temperatures experienced by tiger sharks.

**Shark**	**MaxD (m)**	**MinT (°C)**	**MaxT (°C)**	**RanT (°C)**
T1	248	13.6	27.0	13.4
T2	56	25.0	27.6	2.6
T3	200	15.0	29.0	14.0
T5	483	8.3	28.1	19.8
T6	320	9.6	28.2	18.6
T7	840	4.6	30.0	25.4
T8	848	4.6	29.0	24.4
T9	352	10.6	31.2	20.6
T10	1112	4.0	30.0	26.0
T11	318	10.6	27.4	16.8
T12	592	6.2	27.8	21.6
T13	256	12.2	27.2	15.0
T14	368	9.0	27.0	18.0
T15	400	8.4	27.0	18.6
T16	448	8.0	26.8	18.8
T18	400	9.0	26.6	17.6
T19	392	9.4	27.6	18.2
T20	462	8.1	27.5	19.5

Included is the maximum depth (MaxD), minimum temperature (MinT), maximum temperature (MaxT) and temperature range (RanT) for each tiger shark successfully tracked off northeastern Brazil.

**Figure 2:**
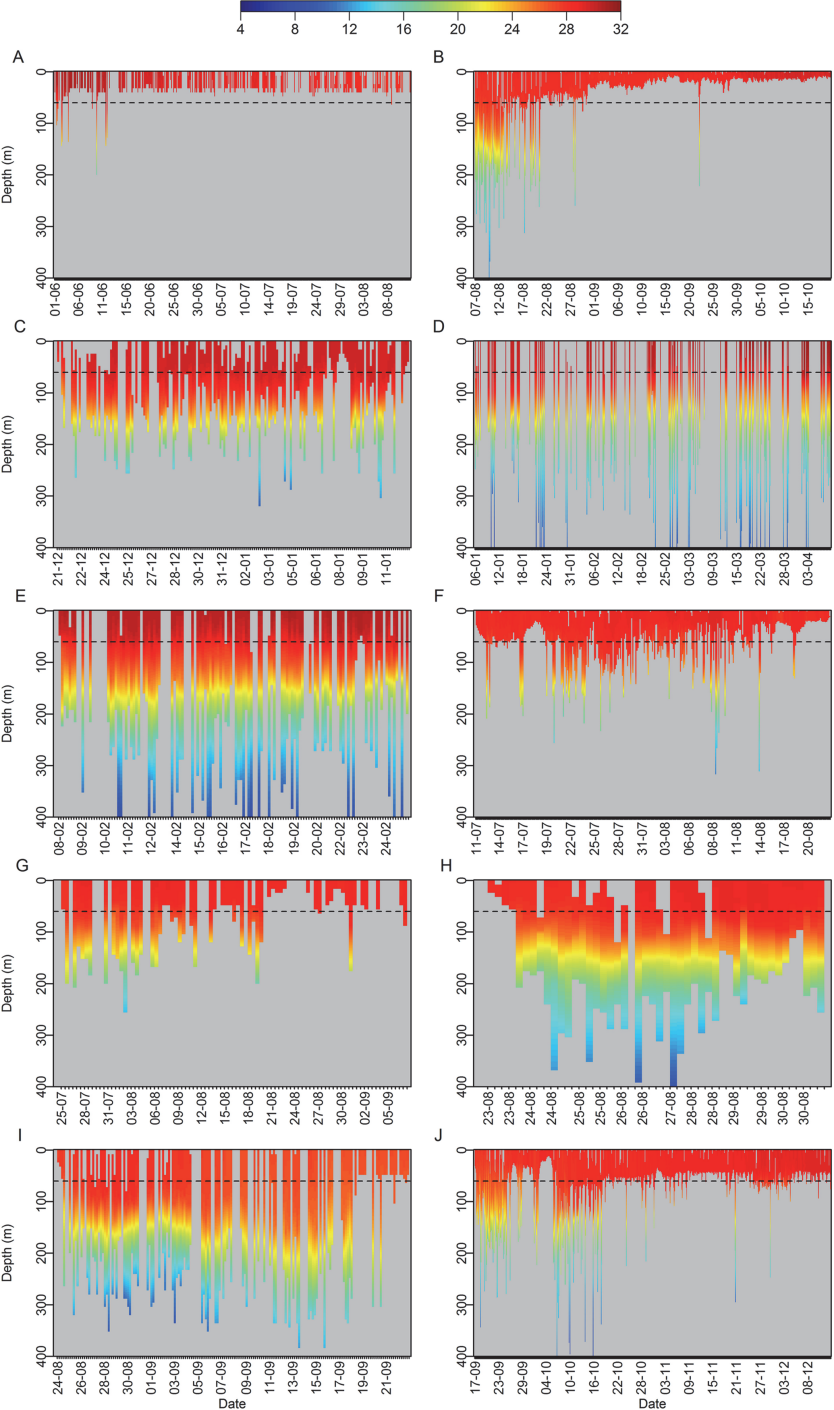
Tiger shark diving behavior. Representative tracks depicting the diving behavior of tiger sharks (A: T3, B: T5, C: T6, D: T7, E: T8, F: T11, G: T13, H: T15, I: T18, J: T20) off northeastern Brazil. The horizontal dashed line represents the 60-m isobath of the shelf break. The color scale represents water temperature (°C).

**Figure 3:**
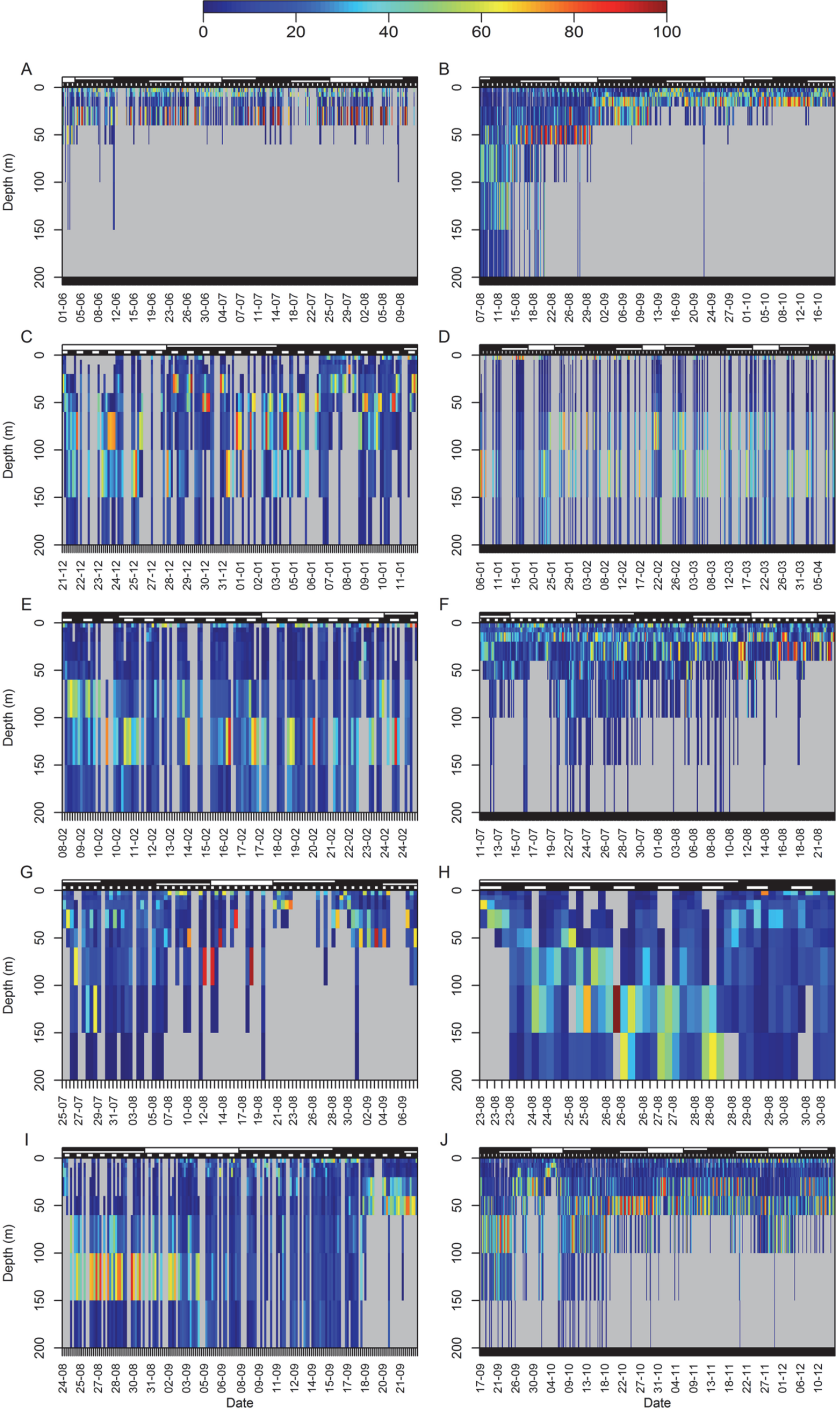
Tiger shark vertical habitat use. Representative tracks depicting depth use in tiger sharks (A: T3, B: T5, C: T6, D: T7, E: T8, F: T11, G: T13, H: T15, I: T18, J: T20) off northeastern Brazil. The proportion of time spent at each depth stratum is informed by the color scale; however, note the different sizes of depth strata, usually larger at greater depths.

Following tag-and-release, most sharks moved into progressively deeper waters, thus implying cross-shelf movement toward the oceanic realm (Figs. 2–3). Thereafter they consistently used a wider portion of the water column and spent a considerable time below the MSL (Fig. 3), but different patterns were observed following an initial time at liberty. Some sharks tended to return to shallower depths, spending most time above the 60-m isobath and only occasionally using deeper waters (i.e. sharks T1, T2, T3, T5, T9, T11 and T14), whereas other sharks continued to make frequent use of waters from the upper thermocline, between the 60- and 150-m isobaths (i.e. sharks T7, T8, T10, T15, T16, T18, T19). Although some sharks did not show preference for any particular depth while using the whole epipelagic and sometimes mesopelagic zones during some periods (Figs. 2–3), alternations between surface- and depth-oriented distributions were repeatedly observed in several individuals regardless of depth range (Fig. 3).

A clear affinity for surface waters was observed in all individuals, with sharks spending, on average, nearly 25% of the global tracking time in waters < 5 m in depth (Table 3). Exceptions were sharks T6, T14, T15, T18 and T20, which spent 9–16% of the tracking time at such depths, contrasting with sharks T2, T9 and T16, which spent more than 40% of the time in surface waters. A decreasing use of progressively deeper water layers was clear, yet sharks T7, T8, T10, T15, T16 and T18 moved at depths > 100 m during at least 20% of the tracking span. Notably, several sharks made little use of subsurface waters, between 5 and 20 m, compared to deeper water layers even though they still exhibited high affinity for surface waters (Table 3). In fact, the results for all sharks combined suggest a slightly positive correlation between the time spent at a specific depth and depths ranging from 5 to 40 m, and a negative correlation at depths > 40 m. Although these percentages correspond to the real proportion of time spent in each depth stratum, they do not directly inform tiger shark preferences for a particular depth stratum due to variability in depth bin size. Depth standardization increased the relative time spent at the surface considerably, up to 35% of the overall time-per-unit-of-depth (TPUD), and eliminated the pattern observed between the 5- and 40-m isobaths, resulting in TPUD decreasing monotonously with depth (S1 Table). Although tiger sharks showed a combined TPUD of 55% in the upper 10 m of the water column and made little use of waters >150 m in depth, they do not seem to prefer any particular depth within the 10- to 60-m as well as within the 60- to 150-m depth intervals. Nevertheless, some intraspecific variability was observed in depth use as some sharks (i.e. sharks T16, T18 and T19) exhibited unimodal surface-oriented distribution whereas other sharks exhibited bimodal distributions with modes at surface and at shallow (20–60 m) depths (i.e. sharks T3 and T20) or extending into deeper waters from the thermocline (i.e. sharks T7, T8, T10 and T15).

**Table 3:** Tiger shark vertical habitat use.

**Shark**	**0–5 m**	**5–10 m**	**10–20 m**	**20–40 m**	**40–60 m**	**60–100 m**	**100–150 m**	**> 150 m**
All	23 (±23)	13 (±16)	15 (±20)	17 (±23)	13 (±21)	9 (±17)	7 (±14)	0.2 (±7)
T1	30 (±14)	20 (±11)	15 (±13)	19 (±11)	9 (±10)	3 (±6)	3 (±6)	0.5 (±1)
T2	52 (±15)	8 (±5)	11 (±9)	17 (±12)	12 (±11)	0	0	0
T3	30 (±22)	26 (±19)	10 (±12)	33 (±35)	1 (±8)	0.1 (±2)	0 (±0.1)	0
T5	19 (±20)	23 (±20)	30 (±28)	11 (±19)	11 (±24)	3 (±11)	3 (±10)	1 (±4)
T6	11 (±15)	3 (±4)	5 (±8)	17 (±21)	23 (±23)	22 (±23)	15 (±18)	3 (±5)
T7	27 (±25)	1 (±2)	1 (±2)	3 (±2)	4 (±4)	23 (±18)	29 (±20)	11 (±10)
T8	25 (±21)	4 (±5)	4 (±6)	5 (±5)	6 (±6)	18 (±14)	27 (±21)	11 (±9)
T9	57 (±33)	14 (±15)	14 (±20)	13 (±24)	2 (±8)	0.4 (±3)	0.2 (±2)	0.1 (±1)
T10	29 (±21)	5 (±4)	7 (±7)	6 (±5)	6 (±6)	24 (±17)	16 (±12)	5 (±6)
T11	21 (±19)	15 (±11)	30 (±21)	20 (±24)	8 (±15)	4 (±8)	1 (±3)	0.2 (±1)
T12	35 (±26)	7 (±6)	10 (±11)	17 (±21)	8 (±10)	12 (±15)	8 (±13)	4 (±9)
T13	24 (±19)	13 (±11)	15 (±16)	17 (±19)	17 (±22)	11 (±21)	3 (±9)	0.2 (±0.3)
T14	12 (±15)	17 (±20)	27 (±25)	25 (±30)	6 (±11)	6 (±12)	5 (±13)	1 (±4)
T15	9 (±15)	5 (±6)	9 (±12)	13 (±14)	11 (±12)	16 (±14)	23 (±21)	14 (±17)
T16	42 (±26)	7 (±5)	6 (±5)	4 (±3)	4 (±3)	10 (±7)	18 (±18)	9 (±13)
T18	15 (±16)	9 (±10)	10 (±12)	12 (±13)	13 (±18)	13 (±12)	22 (±25)	7 (±7)
T19	28 (±22)	7 (±8)	9 (±11)	16 (±20)	15 (±17)	20 (±26)	4 (±9)	0.2 (±1)
T20	16 (±18)	9 (±11)	9 (±11)	21 (±23)	29 (±26)	13 (±21)	3 (±8)	0.2 (±1)

Overall proportion of time (mean ± SD), in raw percentage, spent at each depth stratum for all sharks combined and each shark separately. Note that averages in each row may not always sum 100 due to rounding errors.

The thermal distribution followed a similar trend, with all tiger sharks combined spending 75% of the time swimming in warm (> 26°C) waters from the MSL and only 4% of the time in temperatures below 22°C (Table 4). However, some variability was observed since sharks such as T7, T8, T10, T15, T16 and T18 made a relevant (> 10%) use of temperatures below 22°C, whereas sharks T2, T3, T5, T9, T11, T13, T14, T19 and T20 seldom experienced these temperatures. Also, sharks T7, T9 and T10 differed from the remaining sharks in using waters > 30°C for a period of time. Note should be taken that sharks T2 and T16 transmitted little data and the respective results may not be representative of their overall behavior.

**Table 4:** Tiger shark thermal preferences.

**Shark**	**< 12°C**	**12–18°C**	**18–22°C**	**22–26°C**	**26–30°C**	**> 30°C**
All	0.1 (±1)	1 (±3)	3 (±8)	21 (±32)	74 (±36)	1 (±10)
T1	0	0.1 (±0.2)	1 (±1)	24 (±21)	76 (±21)	0
T2	0	0	0	5 (±22)	95 (±22)	0
T3	0	0	0	3 (±14)	97 (±14)	0
T5	0 (±0.1)	0.3 (±1)	1 (±4)	12 (±27)	86 (±29)	0
T6	0 (±0.3)	1 (±2)	4 (±7)	18 (±22)	78 (±26)	0
T7	1 (±3)	6 (±7)	13 (±10)	34 (±20)	46 (±25)	6 (±14)
T8	1 (±2)	5 (±5)	8 (±9)	39 (±24)	48 (±27)	0
T9	0	0.1 (±0.3)	0.1 (±1)	3 (±11)	97 (±12)	21 (±38)
T10	1 (±2)	4 (±6)	10 (±12)	32 (±19)	53 (±26)	6 (±11)
T11	0 (±0)	0 (±0.2)	0.3 (±2)	7 (±17)	93 (±17)	0
T12	0.2 (±1)	1 (±3)	6 (±15)	39 (±38)	54 (±39)	0
T13	0	0.1 (±0.2)	2 (±5)	19 (±30)	35 (±40)	0
T14	0 (±0.2)	1 (±1)	2 (±7)	15 (±29)	83 (±31)	0
T15	0.1 (±0.3)	4 (±5)	20 (±27)	30 (±26)	46 (±36)	0
T16	0.4 (±1)	3 (±4)	13 (±14)	43 (±28)	41 (±32)	0
T18	0 (±0.1)	2 (±2)	8 (±10)	86 (±17)	5 (±13)	0
T19	0.1 (±0.3)	0.4 (±1)	1 (±1)	74 (±32)	25 (±32)	0
T20	0 (±0.4)	0.3 (±1)	1 (±3)	19 (±32)	80 (±34)	0

Overall proportion of time (mean ± SD), in percentage, spent at each temperature stratum for all sharks combined and each shark separately. Note that the sum of each row may not always equal 100 due to rounding errors.

### Diel and lunar patterns

**Figure 4:**
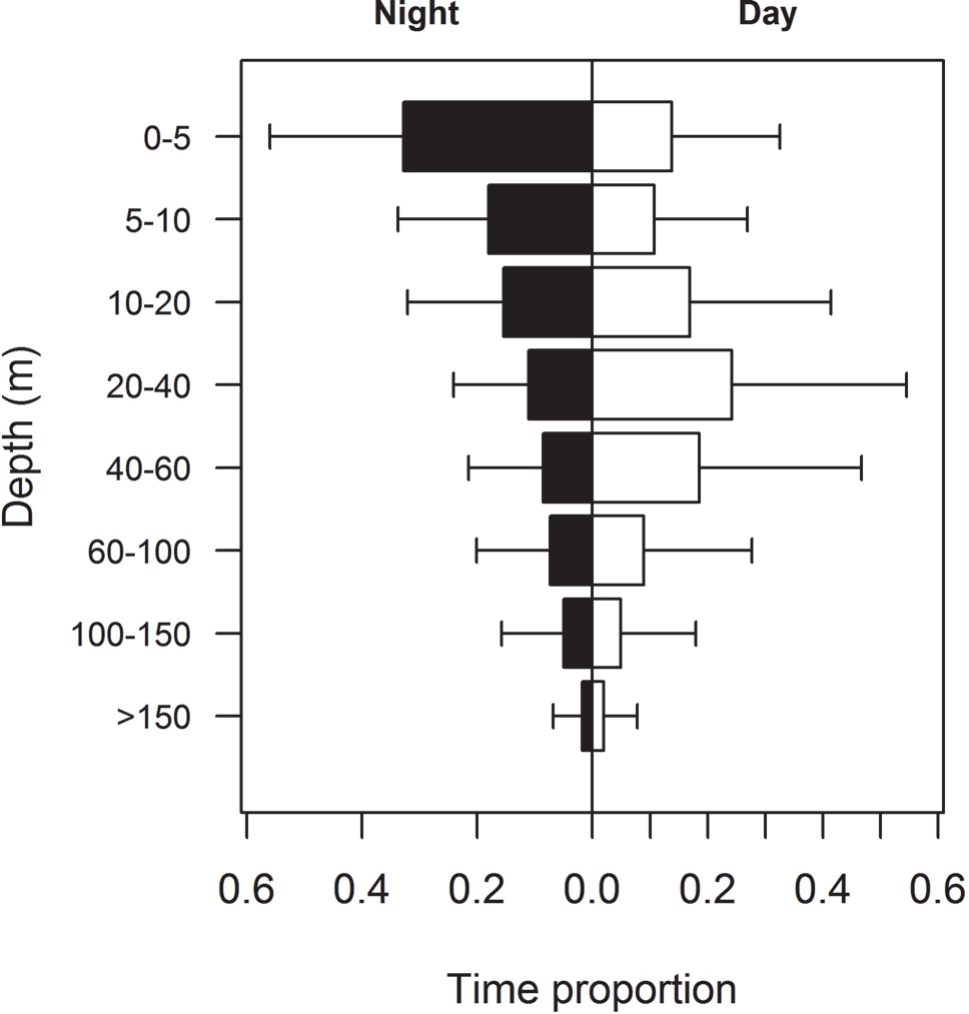
Diel variability in vertical habitat use. Diel behavioral shift in tiger shark depth use, as the proportion of time spent at each depth stratum, with sharks spending more time at the surface during the night time (solid bars) and more time between the 20- and 60-m isobaths during the daytime (blank bars). The error bars represent standard deviations.

Overall, tiger sharks exhibited a diel shift in depth usage by spending, on average, more than twice the time in surface waters (0–10 m) during the night than during the day (Fig. 4). The upper 5 m of the water column were strikingly preferred during the night, whereas in daylight tiger sharks preferred slightly deeper waters, between 10 and 60 m in depth with a mode at 20–40 m. However, higher percentage means were associated with higher data dispersion (Fig. 4). No obvious differences in the time spent at depths below 60 m were observed.

**Figure 5:**
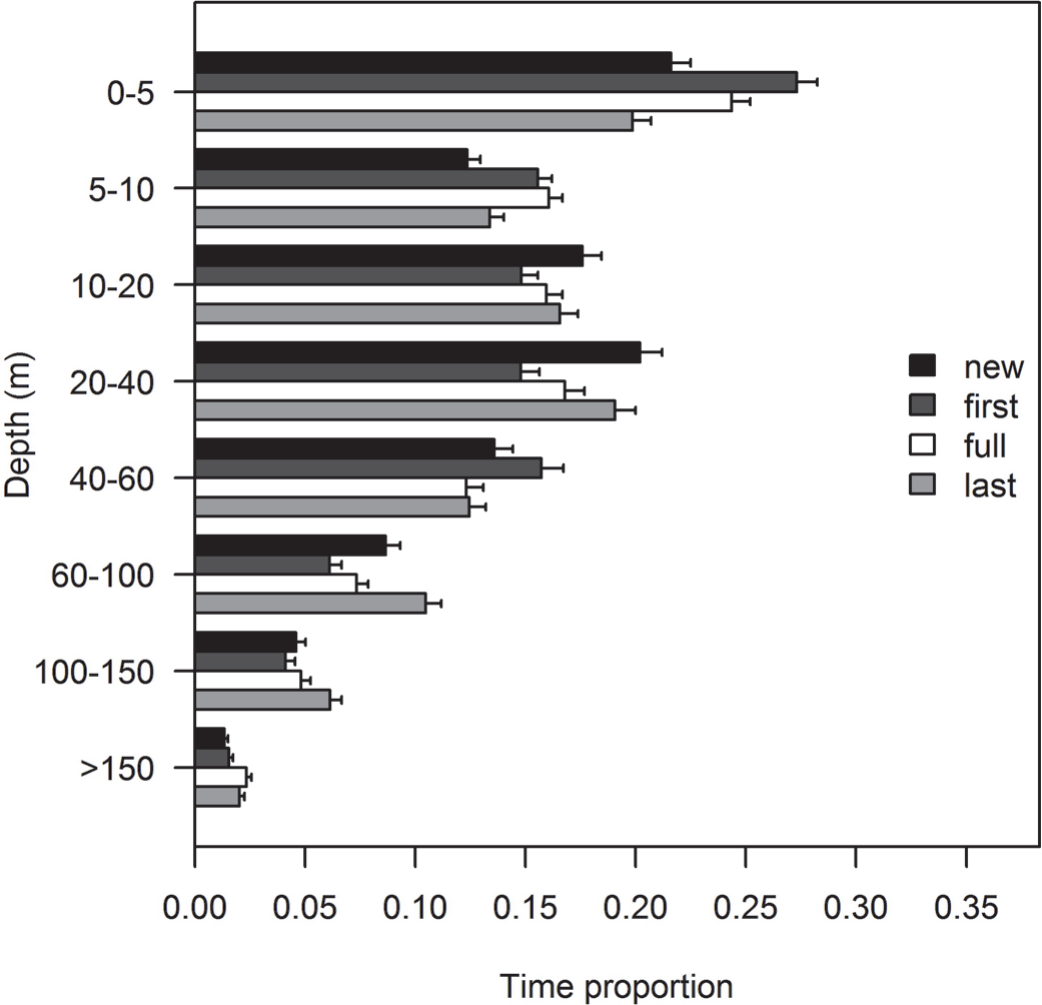
Lunar variability in vertical habitat use. Tiger shark depth use, as the proportion of time spent at each depth stratum, across the four moon phases (new moon, first quarter, full moon and last quarter) of the lunar cycle. The error bars represent standard deviations.

Differences in depth usage throughout the lunar cycle were inconspicuous. Tiger sharks tended to spend more time in surface waters (0–10 m) during the first quarter and full moon phases, whereas they spent more time at depths of 20–40 and 60–100 m during the last quarter and new moon phases (Fig. 5). Yet, differences were generally small, in the order of percent units, and seemingly coherent trends across the water column were disrupted in some depth intervals (e.g. 40–60 m).

### The effect of size and sex

**Figure 6:**
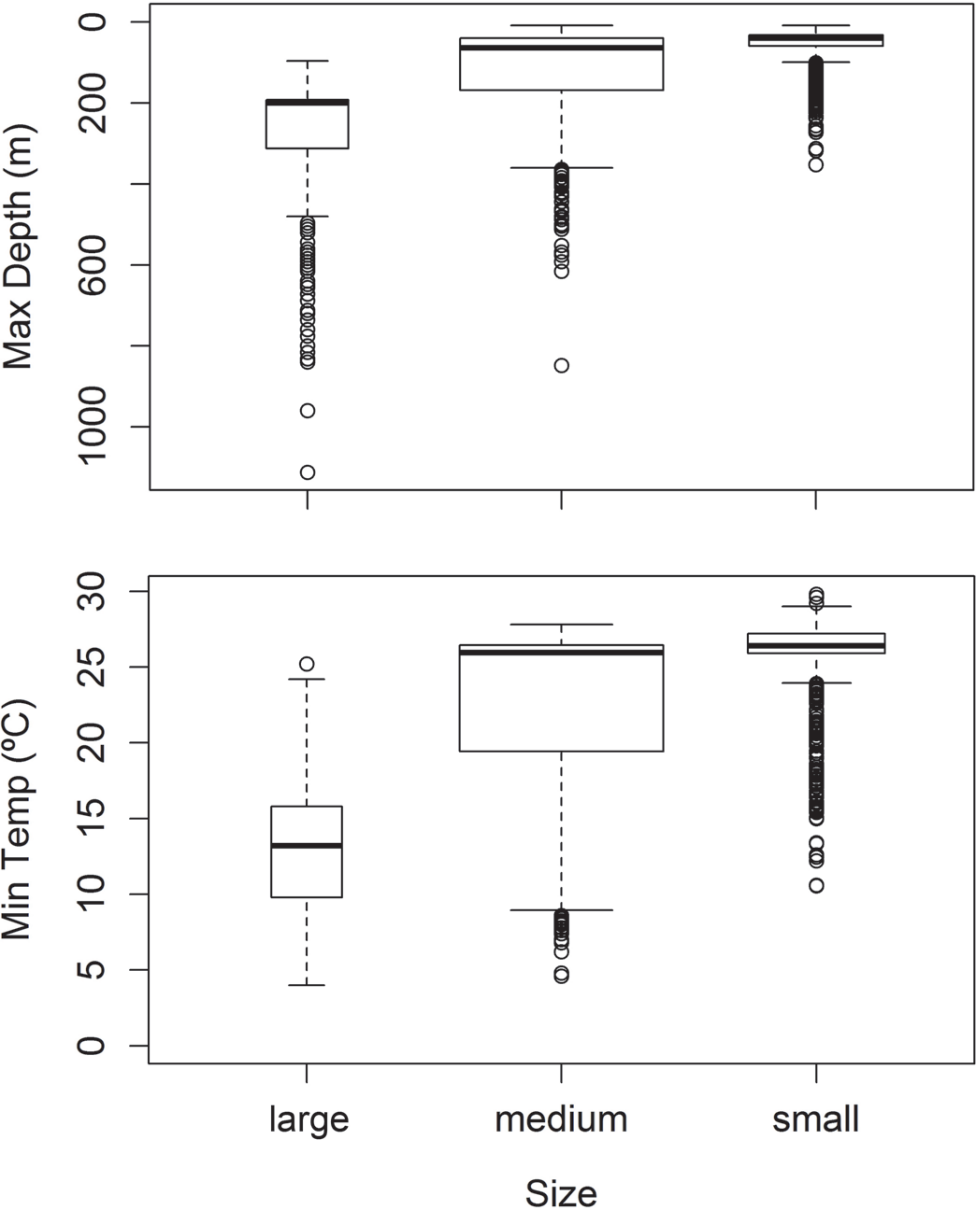
Influence of shark size on diving behavior. Boxplots depicting the distribution of maximum diving depth (top panel) and minimum diving temperature (bottom panel) across three size-classes (large, medium and small) of juvenile tiger sharks. The box represents the first and third quartiles and the bold horizontal bar represents the median, whereas circles represent outliers. The box width is proportional to the logarithm of sample size. A total of 3169 samples were used.

The MDD increased with shark length. The deepest dives performed by small sharks were mainly distributed along the upper ~100 m of the water column (Fig. 6). Compared to small sharks, medium sharks had greater interquartile range and dove into deeper waters but both the first quartile and median were very similar to those of small sharks. Large sharks, however, showed a MDD distribution extending to considerably deeper waters, with the first quartile equaling the median at the 200-m isobath and the last quartile placed at the ~500-m isobath. Dives deeper than 600 m were mostly performed by large sharks. A Kruskal-Wallis test detected significant differences in MDD between size classes (χ^2^ = 874.56, p < 0.001). Likewise, MDT in small tiger sharks showed a narrow distribution mostly within warm waters from the MSL, whereas medium and large sharks were exposed to much lower temperatures (Fig. 6), with significant differences in MDT between size classes being detected (χ^2^ = 708.69, p < 0.001). The MDT in large sharks was particularly wide-ranging, spanning from 4 to ~25°C. No plots were generated for inspecting differences in MDD or MDT between sexes because such relation requires particular caution due to shark length correlating positively with MDD and MDT and the largest sharks in this study being all female. However, Kruskal-Wallis tests applied to a balanced universe encompassing four sharks (i.e. sharks T6, T8, T12 and T15) detected significant differences in both MDD (χ^2^ = 75.66, p < 0.001) and MDT (χ^2^ = 28.63, p < 0.001) between sexes, although they were small in average (MDD ≈ 13 m; MDT ≈ 0.7°C).

**Figure 7:**
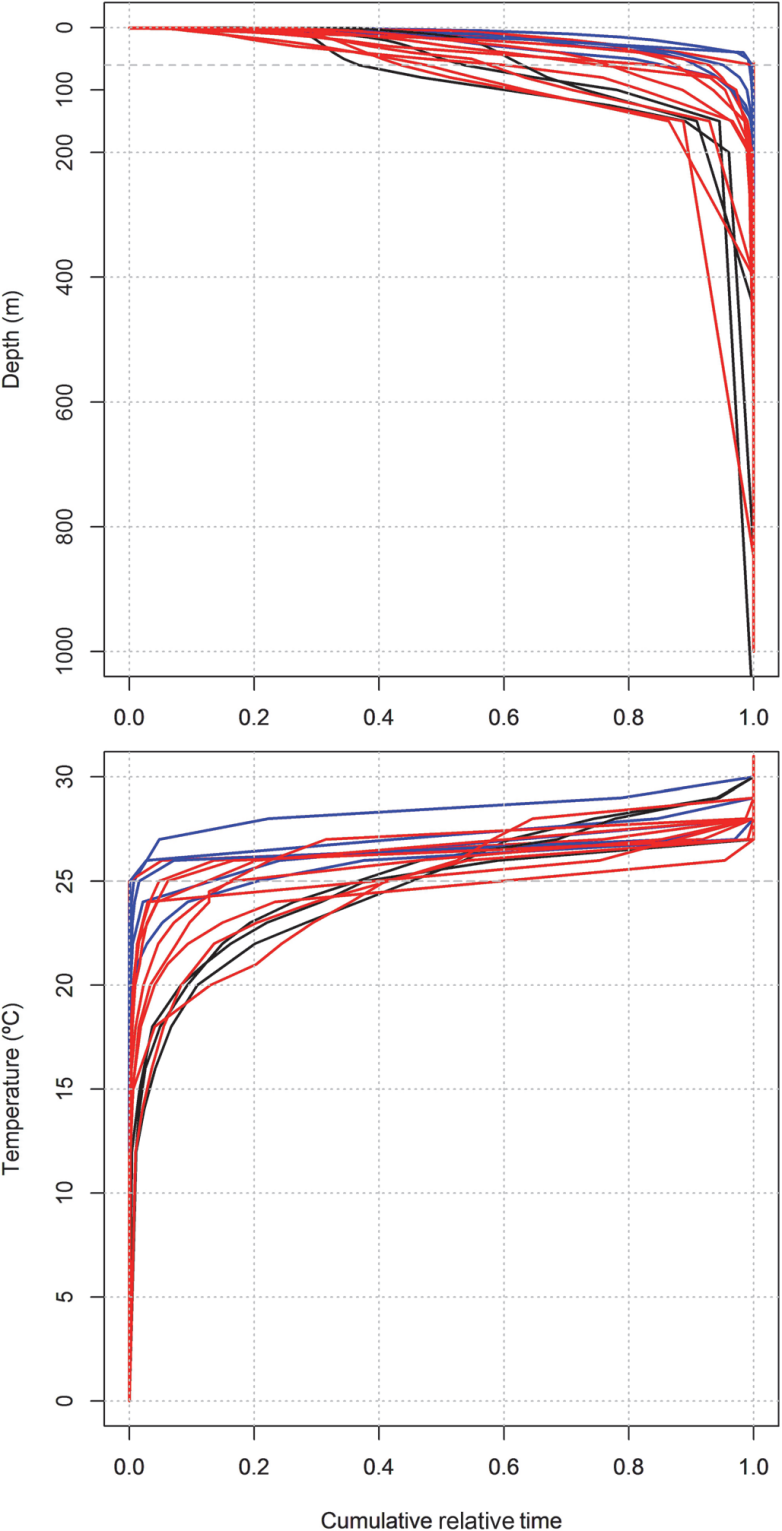
Influence of shark size on depth and temperature use. Cumulative plots of the percentage of time spent by juvenile tiger sharks across consecutive depth (top panel) and temperature (bottom panel) intervals. The separation in blue (for small-sized sharks), red (for medium-sized sharks) and black (for large-sized sharks) points out to an ontogenetic shift in vertical habitat use, with larger sharks spending more time in deeper, colder waters.

The time spent at specific depths through the water column also varied across size classes. Small sharks coherently spent most of the tracking time at depths < 60 m and little or no time at depths > 100 m (Fig. 7). Medium-sized sharks showed some variability in depth use but most individuals differentiated from small-sized sharks by spending more time in deeper waters, which was already noticeable at short cumulative percent times. Large sharks made a considerable use of even deeper waters, although they still spent more than 90% of the tracking time at depths < 200 m. These sharks spent between ~35 and ~65% of their tracking times at depths < 60 m, although a few medium-sized sharks showed a similar trend. Differences in thermal habitat use between size classes were also noticeable. Most small- and medium-sized sharks made little (< 20%) or no use of waters cooler than 25°C, hence they spent most or all time in the MSL (Fig. 7). Such sharks are readily distinguishable because they show small temperature variation across most of the cumulative percent time scale, which results from a small (~5°C) temperature range in the MSL. On the other hand, large-sized and a couple of medium-sized sharks made a substantial use of waters as cold as 15°C and did not show any clear preference for a specific temperature within the 20–30°C range.

### Generalized linear models

A total of 1062 and 802 samples from 15 sharks were used for modeling trends in MDD and MDT, respectively, whereas 824 samples were used for modeling trends in TAS. The Gamma error distribution was selected to model MDD and MDT using identity and log as canonical link functions, respectively, whereas the Binomial error distribution with a logit link function was selected to model TAS.

**Figure 8:**
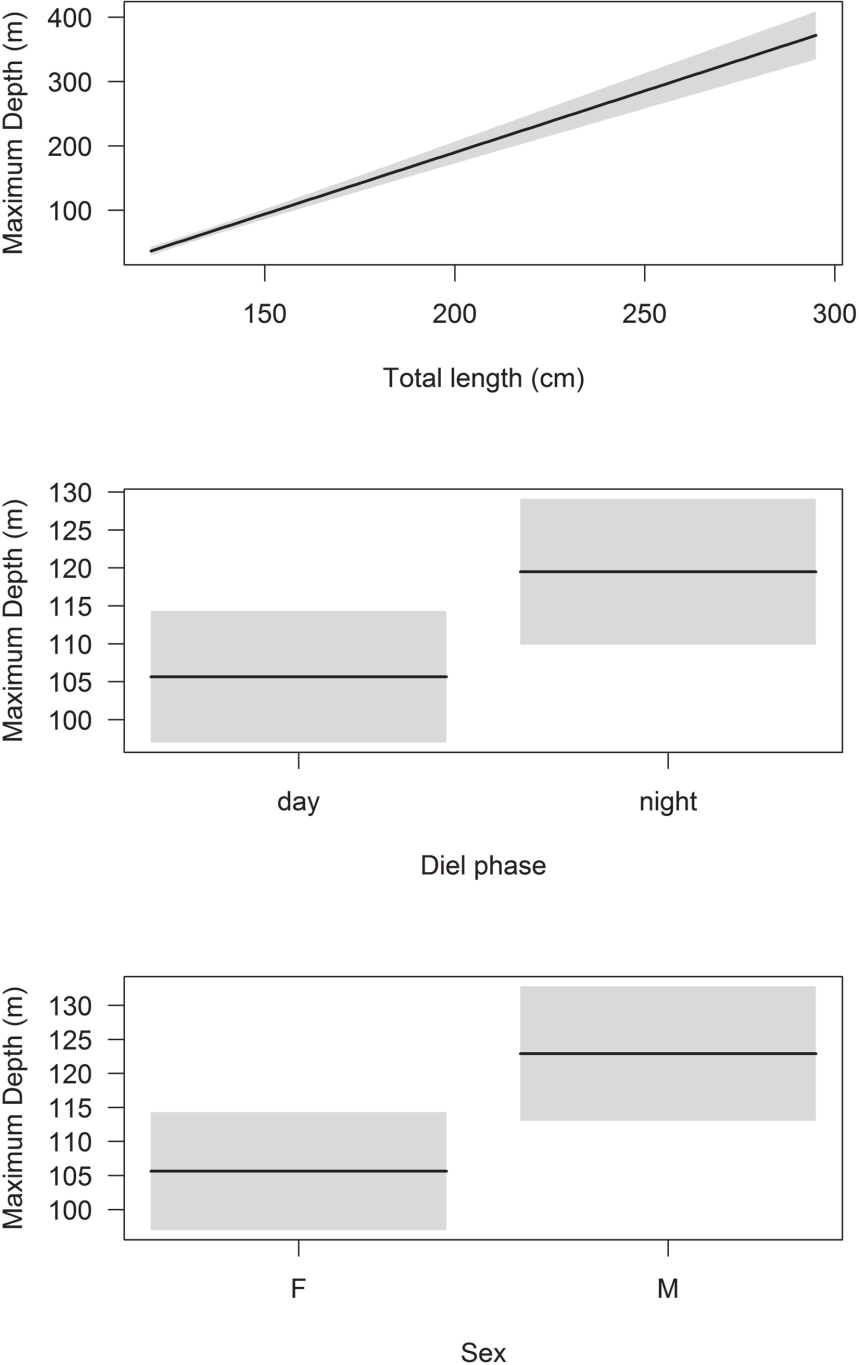
Maximum diving depth models. Generalized linear models showing the effects of shark length (top panel), diel phase (middle panel) and sex (bottom panel) on the maximum diving depth of juvenile tiger sharks. Note the different scales on the y-axes.

**Figure 9:**
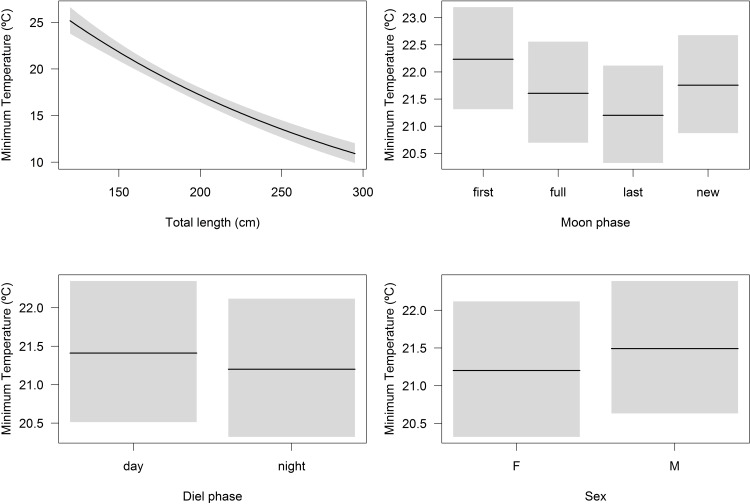
Minimum diving temperature models. Generalized linear models showing the effects of shark length (top-left panel), moon phase (top-right panel), diel phase (bottom-left panel) and sex (bottom-right panel) on the minimum diving temperature of juvenile tiger sharks. Note the different scales on the y-axes.

The GLM output for MDD modeling selected shark length, diel phase and sex as the most important factors, in that same order (S2 Table). The resulting coefficient of dispersion and explained deviance were 0.578 and 41.6%, respectively. MDD increased proportionally to shark length, with small (< 150 cm TL) sharks diving into waters less than 100 m in depth and large (250–300 cm TL) sharks diving into 300–400 m isobaths, which represents a 3- to 4-fold increase in maximum diving depth (Fig. 8). MDD was significantly higher during the night than during the day and it was higher for males compared to females (Fig. 8). The most parsimonious MDT model included all variables (i.e. shark length, lunar and diel cycles, and sex), resulting in a coefficient of dispersion of 0.063 and a total of 47.2% of explained deviance (S2 Table). Significant interactions between shark size, diel cycle and sex, and between shark size and lunar cycle, were all included in the final model. MDT decreased considerably with shark size, with small (< 150 cm TL) sharks experiencing minimum temperatures > 20°C, whereas sharks > 250 cm TL experienced minimum temperatures below 15°C (Fig. 9). The lunar cycle, independently, had a marginally significant effect on MDT, but its interaction with shark size increased the statistical significance of this factor (S2 Table). Tiger shark MDT was slightly higher for the day period and for males compared to the night period and females, but differences should be biologically insignificant (Fig. 9).

**Figure 10:**
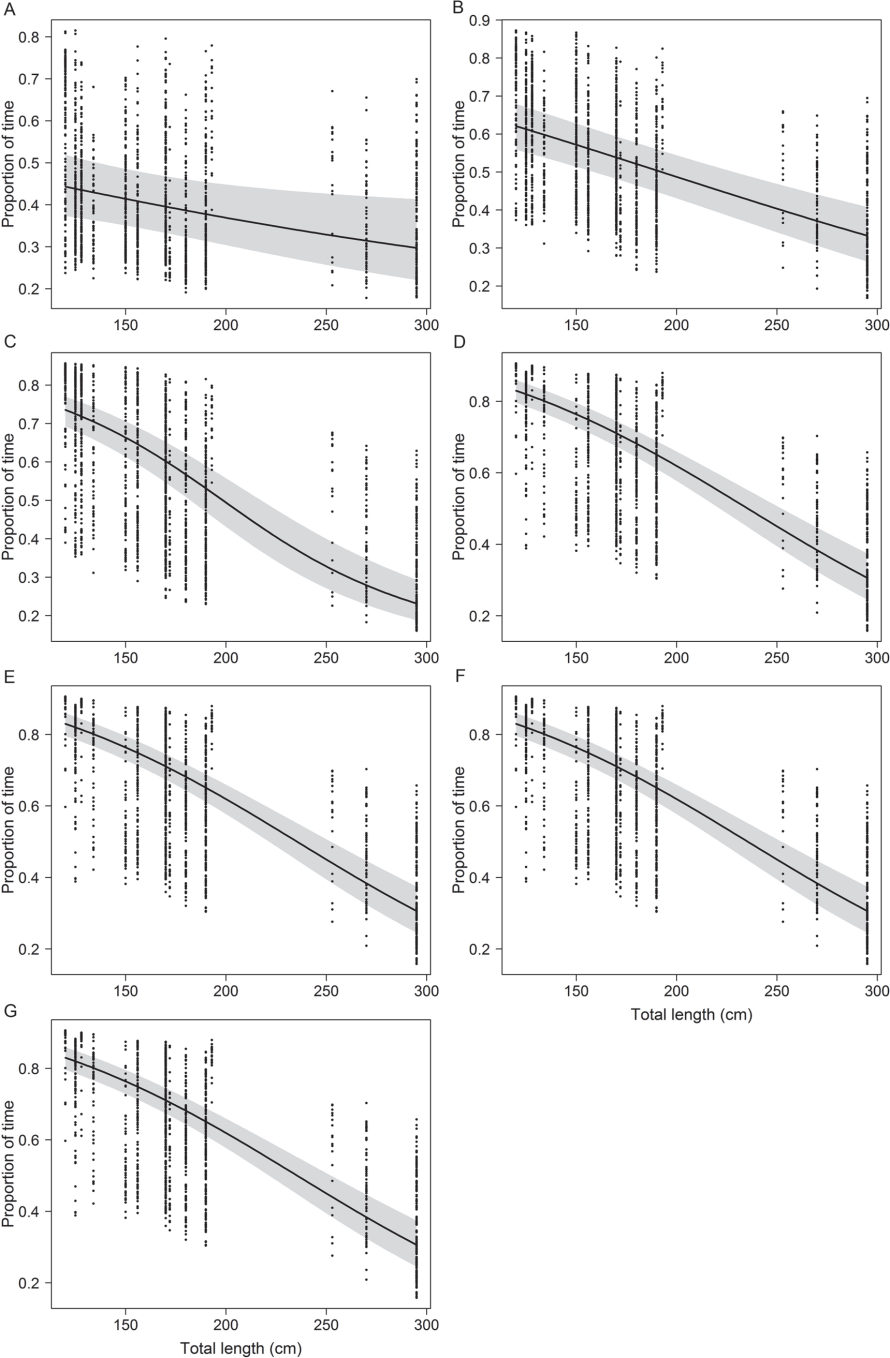
Ontogenetic variability in shallow habitat use. Generalized linear models showing the effect of shark length on the proportion of time spent by tiger sharks in surface waters. Surface waters were defined as the water layer between the sea surface and a given isobath. These isobaths were set to (A) 10 m, (B) 20 m, (C) 40 m, (D) 60 m, (E) 100 m and (F) 150 m. The 5-m isobath was not included because shark length had no significant effect on the time spent above such depth. The black dots represent the empirical data. Note the different scales on the y-axes.

Regarding TAS modeling, some interesting trends were observed across the seven different ranges of surface depth. TAS_[5]_ was influenced by diel phase and sex, with shark length not being selected when surface waters comprised the upper 5 m of the water column only (S3 Table). However, shark length was included in the remaining six models and became the most important or the only factor influencing TAS when the surface water layer was set to a depth of at least 20 m. The diel phase was always included in models TAS_[5]_ through TAS_[40]_, whereas sex was further included in TAS_[100]_. Increasing shark length resulted in a clear decrease in TAS in models TAS_[10]_ through TAS_[150]_, although differences were substantially attenuated in the latter model (Fig. 10). A subtle accentuation in the rate of TAS decrease was visible for sharks larger than ~200 cm TL, particularly in models TAS_[60]_ through TAS_[150]_. Furthermore, a considerable rise in the proportion of deviance explained by the models was verified when surface waters extended to at least the 40-m isobath (S3 Table). However, Q-Q diagnostic plots indicated that models TAS_[40]_ through TAS_[150]_ could not conform to the normality assumption, thus requiring caution for interpreting the outcome of statistical tests. Since the effect of TL was always highly significant (p < 0.001), though, such caveat should be less likely to influence the interpretation of this variable.

## Discussion

The tiger shark is an apex predator in marine trophic webs [1, 40, 47], therefore it may impact community dynamics. The depletion of apex predators may lead to serious ecological damage such as mesopredator releases and trophic cascades [3, 4, 16, 48], and the simulated removal of tiger sharks from a tropical food web has been associated with the greatest changes in the biomass of other *taxa*, whereas the removal of reef sharks, *Carcharhinus* spp., had comparatively small effects [5]. On the other hand, tiger sharks are distinguished from other carcharhinids by their ovoviviparity and their home ranging behavior spanning through large spatiotemporal scales [34, 35, 49] and including a broad variety of marine habitats across both coastal and oceanic realms [33, 50–52]. This lack of specialization poses a number of constraints to management and research efforts aiming at ensuring the conservation of tiger shark populations. For example, unlike several other tropical carcharhinids that use coastal habitats during early life stages to enhance survival and growth [53], young-of-the-year (YOY) tiger sharks seem to be more abundant in offshore waters from the continental shelf and are apparently not confined to specific areas [37]. Such characteristic could preclude conservation strategies based on the protection of specific regions or habitats to promote later recruitment to adult populations. Furthermore, it also restrains the ability to attain an adequate knowledge of tiger shark populations that ensures proper measures are taken to keep them at sustainable levels.

Understanding the dynamics of habitat utilization in tiger sharks throughout their life cycle is required to implement effective management efforts. Juvenile tiger sharks measuring < 200 cm TL occur off Recife from January through September but they become infrequent from October onwards [54]. Small (< 100 cm TL) neonates are present during the first trimester only, and a modal progression in shark size from the first through the third trimesters suggests that YOY tiger sharks use neritic habitats to enhance growth until they attain about 150–200 cm TL, after when they presumably shift to oceanic habitats [54]. Such size class likely corresponds to YOY individuals because tiger sharks are born at 51–90 cm TL [39, 55] and they may exhibit growth rates as high as ~100 cm·y^−1^ during the first year of free-swimming in this region [56]. Although the distribution of juvenile tiger sharks on the Brazilian shelf is generally unknown, satellite telemetry showed that they perform long-distance movements along the continental platform and do not seem to remain in any specific location for protracted periods of time [44, 57]. Therefore, it is likely that YOY tiger sharks are dispersed throughout the neritic province off northeastern Brazil, similarly to early juveniles from US waters [37]. Such distribution provides ready accessibility to a great extension of demersal habitats but it also constraints the vertical range of shark movements due to the bathymetric profile of these waters, which does not happen in the oceanic realm. Assessing patterns in vertical habitat usage at early life stages could thus be most useful to elucidate the intrinsic and extrinsic processes that regulate tiger shark distribution and behavior on the continental shelf and in deep oceanic habitats.

Tiger sharks in this study used a considerable portion of the water column until a maximum depth of 1112 m, a record similar to the deepest tiger shark dive reported off northeast Australia [52], but they moved mostly above the 150-m isobath. Likewise, this species has been reported to mainly use the upper 100 m of the water column although performing periodic dives into deeper waters [32, 49, 58, 59]. Some intraspecific variability in depth distribution was noticed as some sharks showed a striking preference for waters < 60 m in depth whereas others used waters below the 60-m isobath substantially. The continental platform off northeastern Brazil consists of a narrow shelf (63 km average width) that slants smoothly until about the 60-m isobath, where a steep (4 to 20°) slope abruptly starts [60]. Therefore, such variability could partially result from the location of the shark in relation to the shelf. Consistent depth ‘floors’ in tiger shark movements above the thermocline have been previously ascribed to the presence of shallow habitats [49] but in this study the depth of the shelf break coincides with the depth of the thermocline. While this fact could obscure the association of depth ‘floors’ in our dataset with habitat shallowness, the existence of extended periods of time during which some sharks moved exclusively in shallow (< 60 m) waters or in cold waters from the thermocline suggests that bathymetric constraints rather than temperature originated depth ‘floors’ around the 60-m isobath. Notwithstanding, differences in depth usage could also emerge from shifts between surface- and depth-oriented behavioral phases. Tiger sharks from the Caribbean and western North Atlantic showed variability in vertical habitat use, and surface-oriented behaviors were distinguished from ‘bimodal-shallow’ behaviors in which sharks spent comparably less time at the surface and more time around the 20–60 m isobaths [59], a pattern that agrees with the trends observed in this study. Off northeastern Brazil it is plausible to associate such ‘bimodal-shallow’ depth distribution to bottom-oriented behavior due to the depth ‘floors’ imposed by the shelf, but the sharks in [59] were tagged off oceanic islands and some moved through long distances in the pelagic realm where hard substrate is expectedly inexistent at such depths. Other abiotic and biotic factors such as dissolved oxygen concentration [61], the geomagnetic field [62], and prey distribution [63] have been previously associated with shark movements and could be adding complexity to the patterns of vertical habitat use in tiger sharks.

A strong affinity for surface (< 5 m) waters in the neritic as much as in the oceanic provinces is a common trait in tiger sharks described in this study and elsewhere [35, 59, 64]. However, a greater affinity for subsurface (10–20 m) waters and a negligible use of surface (< 10 m) waters in conspecifics off Australia has been reported [58]. Also, tiger sharks off Hawaii tended to exhibit depth—rather than surface-oriented movements [32]. While the outcome of the latter study could be explained by tiger sharks being tracked for the first 19–49 hours at liberty, a period during which tiger sharks could experience post-release stress and tend to move into deeper waters [44], the former is more intriguing although it could be ascribed to regional differences between distinct study areas. Off Brazil, the preference for the upper meters of the water column was further highlighted after depth-bin standardization and might be explained by foraging behavior because tiger sharks prey upon several air-breathing marine *taxa* including turtles, mammals and birds [38, 39]. In agreement, oscillatory, yo-yo diving behavior has been frequently detected in tiger sharks while moving in epipelagic waters [44, 45, 59] and such strategy could promote predatory efficacy by increasing the amount of water volume potentially scanned by a foraging shark. In this perspective, tiger sharks moving on the Brazilian shelf would expectedly exhibit a great surface use and a depth distribution mostly truncated at or shallower than the 60-m isobath, which was identified in several individuals. Also, a few sharks exhibited bimodal depth-standardized distributions which are compatible with depth ‘floors’ from the middle to outer continental shelf (20–60 m), suggesting a higher frequency of bottom-oriented movements within those isobaths. In agreement, tiger shark abundance across the continental shelf off Recife seems to correlate positively with depth [54], similarly to conspecifics from the North Atlantic [65]. Furthermore, the movement trajectories of subadult specimens satellite-tracked off eastern Australia were mostly associated to the shelf edge or mid-shelf areas [66]. On the other hand, some tiger sharks off Brazil moved preferentially in the oceanic realm and showed surface-oriented or bimodal distributions extending into deeper (> 60 m) waters from the thermocline. The consistent use of deeper oceanic habitats could also be related with foraging activities since tiger sharks consume mesopelagic species such as squids [38].

Despite that some sharks showed a relatively uniform behavior during their tracks, significant variability was frequently observed and successfully related to both intrinsic and extrinsic factors. Intraspecific variability in vertical habitat use has been previously reported for this species [59], but in this study we did not test hypothesis at the individual level because we were concerned about the trends that would emerge at the population level during juvenile stages. Additionally, [59] used mostly preadult and adult sharks which may exhibit different trends than the sharks tracked in this study. Juvenile tiger sharks off northeastern Brazil consistently exhibited diel behavioral shifts by spending more time at the surface during the night and more time at deeper (20–60 m) waters during the day, although performing deeper dives during the night. Diel periodicity in tiger shark depth use was also reported for the North Atlantic but opposite trends were observed between individuals [59]. On the other hand, one tiger shark tracked off Hawaii exhibited bottom-oriented behavior in shallow waters during the daytime and deep-diving behavior during the night [41]. This seems to agree with higher detection rates of a number of acoustically-tagged tiger sharks in Hawaiian coastal waters during daytime [33] and higher catch rates of tiger sharks in Shark Bay, Australia, also during daytime [40]. A diversity of intrinsic and extrinsic factors, such as shark age, habitat type, and geographical location, could be interacting with tiger shark behavior and promoting intraspecific variability in diel diving periodicity. The lunar cycle did not have a clear effect on tiger shark behavior, but the time spent at surface tended to be greater during the first quarter and full moon phases, when nocturnal prey are expectedly less cryptic. Also, minimum diving temperatures were lower in the last quarter. Despite the lack of statistical significance, lower tiger shark catch rates around new moon have been reported in recreational fisheries [67], although it remains unclear whether such a trend relates to bait visibility or tiger shark depth behavior. Further research is required to ascertain diel and lunar activity in tiger sharks from different regions and environments in order to clarify the predictability of their behaviors across periodic environmental processes.

More interestingly, intrinsic factors had a considerable influence on tiger shark depth distribution, particularly shark length. An ontogenetic habitat expansion in juvenile tiger sharks off northeastern Brazil has been quantitatively described in this study, with larger sharks accessing deeper, colder habitats. Such new information contributes clearly to clarifying tiger shark spatial ecology in this region. Neonates from the western South Atlantic seem to use mostly shallow habitats from the continental shelf but, as they grow, they tend to use deeper habitats from the outer shelf and slope, eventually becoming less attached to the neritic province and performing long-term incursions through the oceanic realm. Although mature sharks (290–320 cm TL size at maturity; [68, 69]) fall beyond our sampling universe, our model indicates that they use waters of at least ~400 m in depth and may experience temperatures below 10°C routinely. Most published studies on tiger shark movements focus mostly on large subadult and adult specimens thus the trends they report pertain to size-classes not addressed in this study. Nevertheless, and despite that a small number of large juveniles was sampled in this study, there seems to be a coherence between the trends herein documented and empirical data obtained from older individuals elsewhere. Furthermore, an accentuated decrease in the time spent in the upper water column for sharks larger than 200 cm TL corroborates the hypothesis that YOY tiger sharks could be leaving the Brazilian continental shelf into oceanic waters when measuring 150–200 cm TL [54], possibly to forage in deeper oceanic habitats. Although early juveniles did use deep oceanic waters during their tracks, they mostly preferred shallow habitats whereas larger juveniles used more diversified, colder habitats. Thermal inertia could be restraining the use of deep oceanic habitats in small YOY tiger sharks with low body mass [70], which is sustained by a virtually linear relation between shark length and minimum diving temperature. Positive correlations between body size and maximum depth have been reported for other shark species such as the mako shark, *Isurus oxyrinchus* [71], whereas smaller blue sharks, *Prionace glauca*, were reported to dive deeper than larger ones [63]. In the tiger shark, ontogenetic habitat expansion coupled with ontogenetic dietary shifts could contribute to widen its ecological niche and enhance its generalist behavior because it would allow sharks to forage in different habitats comprising previously inaccessible prey. Regardless, a similar use of surface (< 5 m) waters throughout ontogeny suggests surface-oriented behavior to be a persistent trait in tiger sharks of different ages. Sex was another likely factor influencing depth distribution but differences observed between sexes were generally small and should have little biological meaning, at least in regard to juvenile tiger sharks.

## Conclusions

Tiger sharks are believed to be relatively resilient to habitat degradation because they are generalist feeders and have low habitat specialization [72]. Despite that neonate tiger sharks are potentially more exposed to human pressure during the first months after birth, when they make most use of shallower nearshore habitats, they seem to disperse into oceanic waters and use deeper pelagic habitats within one year after birth. Such an early plasticity in habitat use could provide access to previously unavailable prey, which agrees with the generalist behavior of this species. Indeed, a wide variety of prey and habitats could endow tiger sharks with a higher resilience against deleterious processes, but their populations are still exposed to considerable fishing pressure in both coastal and pelagic environments [13, 51, 65, 73]. Off northeastern Brazil, about 25% of the tiger sharks tagged and released have been caught in relatively little time at liberty either by artisanal fishermen operating gillnets from small boats or by presumably pelagic longliners operating around oceanic seamounts [44], suggesting that a significant proportion of individuals is being removed from the population by fisheries. Previous studies have shown some of the intricacies of tiger shark spatial ecology, having identified traits that regulate migratory behavior such as the reproductive cycle [74], use of seasonal foraging grounds [40, 75], and fidelity to specific locations or reefs [52, 58]. The development of accurate 3-dimensional ontogenetic models of tiger shark movements and distribution will further benefit management strategies and contribute to the conservation of their populations. However, additional research on tiger shark movements across large spatial and temporal scales is required before such goal is achieved, particularly in light of the considerable variability in tiger shark behavior.

## Supporting Information

S1 Table Tiger shark depth preferences. Overall proportion of time (mean ± SD), in percentage, spent at each depth stratum for all sharks combined and each shark separately. Data has been standardized by depth unit (m) because depth strata have unequal sizes. Note that averages in each row may not always sum 100 due to rounding errors.
(DOCX)

S2 Table Tiger shark diving behavior. Results of generalized linear models for the effects of shark length (TL), sex, diel cycle (Diel) and lunar cycle (Moon) on the response (Resp) variable, i.e. maximum diving depth (MDD) and minimum diving temperature (MDT). Included are the parameter estimates, 95% confidence intervals (C.I.), standard errors (StErr), the result of the t statistic (t-stat) and the corresponding p-value.
(DOCX)

S3 Table Tiger shark shallow habitat use. Results of generalized linear models for the effects of shark length (TL), sex, diel cycle (Diel) and lunar cycle (Moon) on the proportion of time spent in surface waters. Surface waters were defined as the water layer between the sea surface and a given surface layer depth (SLD). Included are the parameter estimates, 95% confidence intervals (C.I.), standard errors (StErr), the result of the z statistic (z-stat), the corresponding p-value, and the percentage of deviance explained by the model (Dev.Exp.).
(DOCX)
