# Supplementary material for: Vertical Movement Patterns and Ontogenetic Niche Expansion in the Tiger Shark, Galeocerdo cuvier
Source: PLoS One. 2015 Jan 28;10(1):e0116720. doi: 10.1371/journal.pone.0116720 (PMC4309595; doi:10.1371/journal.pone.0116720)
Supplement: S1 Table — Overall proportion of time (mean ± SD), in percentage, spent at each depth stratum for all sharks combined and each shark separately. Data has been standardized by depth unit (m) because depth strata have unequal sizes. Note that averages in each row may not always sum 100 due to rounding errors. (DOCX) [file pone.0116720.s001.docx]

**S1 Table.** **Tiger shark depth preferences.** Overall proportion of time (mean ± SD), in percentage, spent at each depth stratum for all sharks combined and each shark separately. Data has been standardized by depth unit (m) because depth strata have unequal sizes. Note that averages in each row may not always sum 100 due to rounding errors.

| Shark | 0−5 m | 5−10 m | 10−20 m | 20−40 m | 40−60 m | 60−100 m | 100−150 m | > 150 m |
| --- | --- | --- | --- | --- | --- | --- | --- | --- |
| All | 35 (±27) | 20 (±18) | 14 (±18) | 12 (±19) | 11 (±19) | 5 (±13) | 4 (±11) | 0.3 (±2) |
| T1 | 43 (±15) | 29 (±11) | 12 (±9) | 9 (±7) | 5 (±8) | 1 (±3) | 0.8 (±2) | 0.1 (±0.2) |
| T2 | 71 (±14) | 11 (±7) | 8 (±7) | 6 (±5) | 4 (±5) | 0 | 0 | 0 |
| T3 | 38 (±24) | 32 (±21) | 7 (±10) | 22 (±31) | 0.8 (±5) | 0 (±0.4) | 0 (±0.1) | 0 |
| T5 | 25 (±22) | 30 (±22) | 25 (±26) | 7 (±14) | 9 (±22) | 2 (±9) | 2 (±7) | 0.2 (±1) |
| T6 | 24 (±27) | 7 (±8) | 7 (±8) | 13 (±17) | 25 (±26) | 15 (±22) | 8 (±15) | 1 (±2) |
| T7 | 58 (±31) | 3 (±3) | 2 (±2) | 2 (±2) | 4 (±6) | 15 (±17) | 15 (±17) | 0.7 (±1) |
| T8 | 51 (±30) | 10 (±12) | 6 (±7) | 3 (±3) | 4 (±4) | 10 (±12) | 15 (±22) | 0.7 (±1) |
| T9 | 64 (±31) | 17 (±18) | 10 (±16) | 8 (±19) | 1 (±5) | 0.1 (±1) | 0 (±0.5) | 0 (±0.1) |
| T10 | 56 (±22) | 12 (±8) | 8 (±8) | 4 (±3) | 5 (±6) | 10 (±10) | 5 (±8) | 0.2 (±0.2) |
| T11 | 30 (±21) | 24 (±13) | 27 (±20) | 13 (±20) | 5 (±13) | 1 (±3) | 0.2 (±0.9) | 0 (±0.1) |
| T12 | 55 (±30) | 11 (±10) | 10 (±12) | 11 (±17) | 5 (±8) | 5 (±9) | 3 (±11) | 0.3 (±1) |
| T13 | 38 (±20) | 20 (±13) | 13 (±13) | 10 (±13) | 12 (±20) | 6 (±16) | 1 (±4) | 0 (±0.1) |
| T14 | 19 (±17) | 26 (±20) | 25 (±22) | 19 (±26) | 5 (±10) | 3 (±7) | 3 (±7) | 0.3 (±1) |
| T15 | 21 (±23) | 13 (±11) | 12 (±13) | 10 (±9) | 11 (±13) | 10 (±13) | 18 (±24) | 5 (±10) |
| T16 | 63 (±27) | 13 (±9) | 7 (±6) | 2 (±2) | 2 (±2) | 4 (±4) | 7 (±11) | 2 (±3) |
| T18 | 32 (±25) | 18 (±16) | 11 (±12) | 8 (±10) | 10 (±14) | 8 (±11) | 13 (±21) | 1 (±2) |
| T19 | 46 (±28) | 12 (±11) | 9 (±10) | 10 (±17) | 10 (±14) | 10 (±19) | 1 (±4) | 0 (±0) |
| T20 | 28 (±23) | 16 (±14) | 9 (±10) | 15 (±19) | 23 (±25) | 7 (±16) | 1 (±5) | 0 (±0) |
