# Supplementary material for: Vertical Movement Patterns and Ontogenetic Niche Expansion in the Tiger Shark, Galeocerdo cuvier
Source: PLoS One. 2015 Jan 28;10(1):e0116720. doi: 10.1371/journal.pone.0116720 (PMC4309595; doi:10.1371/journal.pone.0116720)
Supplement: S2 Table — Results of generalized linear models for the effects of shark length (TL), sex, diel cycle (Diel) and lunar cycle (Moon) on the response (Resp) variable, i.e. maximum diving depth (MDD) and minimum diving temperature (MDT). Included are the parameter estimates, 95% confidence intervals (C.I.), standard errors (StErr), the result of the t statistic (t-stat) and the corresponding p-value. (DOCX) [file pone.0116720.s002.docx]

**S2 Table. Tiger shark diving behavior.** Results of generalized linear models for the effects of shark length (TL), sex, diel cycle (Diel) and lunar cycle (Moon) on the response (Resp) variable, i.e. maximum diving depth (MDD) and minimum diving temperature (MDT). Included are the parameter estimate, 95% confidence intervals (C.I.), standard errors (StErr), the result of the t statistic (t-stat) and the corresponding p-value.

| Resp | Final Model | Term | Estimate | 95% C.I. | StErr | t-stat | Pr(>│t│) |
| --- | --- | --- | --- | --- | --- | --- | --- |
| MDD | TL + Diel + Sex |  |  |  |  |  |  |
|  |  | Intercept | -193.32 | [-221.01, -166.53] | 14.81 | -13.055 | < 0.001 |
|  |  | TL | 1.92 | [1.72, 2.13] | 0.11 | 17.083 | < 0.001 |
|  |  | Diel_night_ | 13.85 | [4.73, 23.35] | 4.69 | 2.953 | 0.003 |
|  |  | Sex_male_ | 17.27 | [7.96, 27.0] | 5.33 | 3.238 | 0.001 |
| MDT | TL*Diel*Sex + TL*Moon |  |  |  |  |  |  |
|  |  | Intercept | 52.34 | [46.17, 59.34] | 1.07 | 61.158 | < 0.001 |
|  |  | TL | 0.99 | [0.99, 1.00] | 1.00 | -15.273 | < 0.001 |
|  |  | Diel_night_ | 0.79 | [0.70, 0.89] | 1.06 | -3.840 | < 0.001 |
|  |  | Sex_male_ | 0.71 | [0.51, 0.99] | 1.17 | -2.149 | 0.032 |
|  |  | Moon_full_ | 1.16 | [1.00, 1.34] | 1.08 | 1.999 | 0.045 |
|  |  | Moon_last_ | 1.08 | [0.93, 1.26] | 1.08 | 1.024 | 0.306 |
|  |  | Moon_new_ | 0.94 | [0.81, 1.09] | 1.08 | -0.843 | 0.399 |
|  |  | TL*Diel_night_ | 1.00 | [1.00, 1.00] | 1.00 | 4.588 | < 0.001 |
|  |  | TL*Sex_male_ | 1.00 | [1.00, 1.00] | 1.00 | 2.733 | 0.007 |
|  |  | Diel_night_*Sex_male_ | 1.85 | [1.17, 2.93] | 1.25 | 2.798 | 0.005 |
|  |  | TL*Moon_full_ | 1.00 | [1.00, 1.00] | 1.00 | -2.733 | 0.006 |
|  |  | TL*Moon_last_ | 1.00 | [1.00, 1.00] | 1.00 | -1.837 | 0.066 |
|  |  | TL*Moon_new_ | 1.00 | [1.00, 1.00] | 1.00 | 0.636 | 0.525 |
|  |  | TL*Diel_night_*Sex_male_ | 1.00 | [0.99, 1.00] | 1.00 | -3.182 | 0.002 |
