# Supplementary material for: Vertical Movement Patterns and Ontogenetic Niche Expansion in the Tiger Shark, Galeocerdo cuvier
Source: PLoS One. 2015 Jan 28;10(1):e0116720. doi: 10.1371/journal.pone.0116720 (PMC4309595; doi:10.1371/journal.pone.0116720)
Supplement: S3 Table — Results of generalized linear models for the effects of shark length (TL), sex, diel cycle (Diel) and lunar cycle (Moon) on the proportion of time spent in surface waters. Surface waters were defined as the water layer between the sea surface and a given surface layer depth (SLD). Included are the parameter estimates, 95% confidence intervals (C.I.), standard errors (StErr), the result of the z statistic (z-stat), the corresponding p-value, and the percentage of deviance explained by the model (Dev.Exp.). (DOCX) [file pone.0116720.s003.docx]

**S3 Table. Tiger shark shallow habitat use.** Results of generalized linear models for the effects of shark length (TL), sex, diel cycle (Diel) and lunar cycle (Moon) on the proportion of time spent in surface waters. Surface waters were defined as the water layer between sea surface and a given surface layer depth (SLD). Included are the parameter estimate, 95% confidence intervals (C.I.), standard errors (StErr), the result of the z statistic (z-stat), the corresponding p-value, and the percentage of deviance explained by the model (Dev.Exp.).

| SLD | Term | Estimate | 95% C.I. | StErr | z-stat | Pr(>│z│) | Dev. Exp. |
| --- | --- | --- | --- | --- | --- | --- | --- |
| 5 |  |  |  |  |  |  | 20.2% |
|  | Intercept | 0.21 | [0.17, 0.26] | 0.54 | -9.188 | < 0.001 |  |
|  | Diel_night_ | 0.73 | [0.66, 0.79] | 0.54 | 5.847 | < 0.001 |  |
|  | Sex_male_ | 0.36 | [0.29, 0.44] | 0.54 | -3.382 | < 0.001 |  |
| 10 |  |  |  |  |  |  | 22.3% |
|  | Intercept | 0.61 | [0.47, 0.74] | 0.57 | 1.569 | 0.117 |  |
|  | Diel_night_ | 0.72 | [0.66, 0.78] | 0.54 | 6.325 | < 0.001 |  |
|  | TL | 0.50 | [0.50, 0.50] | 0.50 | -4.709 | < 0.001 |  |
|  | Sex_male_ | 0.37 | [0.30, 0.44] | 0.54 | -3.330 | < 0.001 |  |
| 20 |  |  |  |  |  |  | 19.0% |
|  | Intercept | 0.81 | [0.72, 0.88] | 0.56 | 5.587 | < 0.001 |  |
|  | TL | 0.50 | [0.50, 0.50] | 0.50 | -6.962 | < 0.001 |  |
|  | Diel_night_ | 0.69 | [0.63, 0.75] | 0.54 | 5.459 | < 0.001 |  |
| 40 |  |  |  |  |  |  | 28.8% |
|  | Intercept | 0.96 | [0.92, 0.98] | 0.58 | 9.017 | < 0.001 |  |
|  | TL | 0.50 | [0.50,0.50] | 0.50 | -8.649 | < 0.001 |  |
|  | Diel_night_ | 0.61 | [0.53, 0.68] | 0.54 | 2.666 | 0.008 |  |
|  | Sex_male_ | 0.99 | [0.86, 1.00] | 0.80 | 3.184 | 0.001 |  |
|  | TL* Sex_male_ | 0.49 | [0.49, 0.50] | 0.50 | -3.303 | 0.001 |  |

**S3 Table.** Continued.

| SLD | Term | Estimate | 95% C.I. | StErr | z-stat | Pr(>│z│) | Dev. Exp. |
| --- | --- | --- | --- | --- | --- | --- | --- |
| 60 |  |  |  |  |  |  | 35.3% |
|  | Intercept | 0.99 | [0.98, 1.00] | 0.58 | 14.34 | < 0.001 |  |
|  | TL | 0.50 | [0.49, 0.50] | 0.50 | -11.35 | < 0.001 |  |
| 100 |  |  |  |  |  |  | 42.1% |
|  | Intercept | 1.00 | [0.99, 1.00] | 0.64 | 9.408 | < 0.001 |  |
|  | TL | 0.50 | [0.49, 0.50] | 0.50 | -7.177 | < 0.001 |  |
|  | Sex_male_ | 1.00 | [0.96, 1.00] | 0.95 | 2.955 | 0.003 |  |
|  | TL* Sex_male_ | 0.49 | [0.48, 0.49] | 0.50 | -3.153 | 0.001 |  |
| 150 |  |  |  |  |  |  | 30.1% |
|  | Intercept | 1.00 | [0.99, 1.00] | 0.69 | 8.378 | < 0.001 |  |
|  | TL | 0.50 | [0.49, 0.50] | 0.50 | -4.615 | < 0.001 |  |
